# An image inpainting-based data augmentation method for improved sclerosed glomerular identification performance with the segmentation model EfficientNetB3-Unet

**DOI:** 10.1038/s41598-024-51651-1

**Published:** 2024-01-10

**Authors:** Songping He, Yi Zou, Bin Li, Fangyu Peng, Xia Lu, Hui Guo, Xin Tan, Yanyan Chen

**Affiliations:** 1 Digital Manufacturing Equipment National Engineering Research Center, Huazhong University of Science and Technology, Wuhan, China; 2 National NC System Engineering Research Center, Huazhong University of Science and Technology, Wuhan, China; 3 Key Laboratory of Organ Transplantation of Ministry of Education, Institute of Organ Transplantation, Tongji Hospital, Tongji Medical College, National Health Commission and Chinese Academy of Medical Sciences, Huazhong University of Science and Technology, Wuhan, China; 4 Wuhan Intelligent Equipment Industrial Institute Co Ltd, Wuhan, China; 5 Department of Information Management, Tongji Hospital, Tongji Medical College, Huazhong University of Science and Technology, Wuhan, China

**Keywords:** Kidney, Biomedical engineering

## Abstract

The percent global glomerulosclerosis is a key factor in determining the outcome of renal transfer surgery. At present, the rate is typically computed by pathologists, which is labour intensive and nonstandardized. With the development of Deep Learning (DL), DL-based segmentation models can be used to better identify and segment normal and sclerosed glomeruli. Based on this, we can better quantify percent global glomerulosclerosis to reduce the discard rate of donor kidneys. We used 51 whole slide images (WSIs) from different institutions that are publicly available on the internet. However, the number of sclerosed glomeruli is much smaller than that of normal glomeruli in different WSIs, which can reduce the effectiveness of Deep Learning. For better sclerosed glomerular identification and segmentation performance, we modified and trained a GAN (generative adversarial network)-based image inpainting model to obtain more synthetic sclerosed glomeruli. Our proposed inpainting method achieved an average SSIM (Structural Similarity) of 0.8086 and an average PSNR (Peak Signal-to-Noise Ratio) of 22.8943 dB in the area of generated sclerosed glomeruli. We obtained sclerosed glomerular segmentation performance improvement by adding synthetic sclerosed glomerular images and achieved the best Dice of glomerular segmentation in different test sets based on the modified Unet model.

## Introduction

There are a large number of patients in need of kidney transplantation waiting for kidney donors, and this demand is still growing^1^. Meanwhile, many studies have shown that chronic damage to donor kidney biopsy specimens is closely related to transplant outcomes, so approximately 17–20% of collected donor kidneys need to be discarded after pathologist evaluation^2^. Additionally, in daily practice, often due to the urgency of time, different pathologists will have subjective biases when evaluating sections, potentially resulting in unnecessary discarding of organs. We need to minimize the occurrence of this discarding due to the shortage of kidney donors.

In kidney transplant evaluation, there are many indicators to consider, among which the percent of global glomerulosclerosis is considered to be the entry point for kidney transplantation^3^. Due to the large number of glomeruli, the assessment of percent global glomerulosclerosis is very time-consuming and causes poor reproducibility among pathologists. The professional knowledge requirements of pathologists are high, and human error easily occurs. Therefore, automatic image processing methods that can accurately detect and classify glomeruli are needed.

Recently, due to the strong feature extraction ability of Deep Learning, an increasing number of studies have begun to use it to detect or segment objects in pathological images. In the imaging task, CNNs in particular are widely used. Unet, introduced by Ronneberger et al*.* based on CNN^4^, has proven to be useful in many tasks of tissue image segmentation and classification.

However, the performance of Deep Learning often depends on the quantity and quality of the training data. The acquisition of medical images involves the privacy of patients and requires the annotation of experts, so it is relatively difficult to obtain the training data of medical images. Meanwhile, in the publicly available data for glomerular studies, the number of sclerosed glomeruli is much smaller than that of normal glomeruli. Class imbalance can bring difficulties to Deep Learning.

In our study, we proposed a GAN-based image inpainting framework to generate more new sclerosed glomeruli from masks. Innovatively, newly generated sclerosed glomeruli were obtained based on the diverse shapes of the masks and the surrounding contextual information. Furthermore, we improved the segmentation network based on Unet and trained the model by combining the original data with new synthetic data. We realized the automatic segmentation and classification of normal and globally sclerosed glomeruli in digital pathological sections.

## Related research

### GAN and medical image generation

Since Ian Goodfellow proposed GAN in 2014, it has become possible to generate realistic images by designing the game process of the generator and discriminator^5^. Because of their powerful data generation capabilities, an increasing number of GANs have been used in the generation of pathological and medical images to perform data augmentation. In^6^, the combination of VAE and StyleGAN was proposed. The network generated the hidden code of the image through VAE as the input of StyleGAN to generate realistic cell images. In^7^, a medical image augmentation method, namely, a texture-constrained multichannel progressive generative adversarial network (TMP-GAN) was proposed. In^8^, Lei et al. proposed a lesion attention conditional generative adversarial network (LACGAN) to synthesize retinal images with realistic lesion details to improve the training of the disease detection model. Amirrajab, S. et al. proposed a novel framework consisting of image segmentation and synthesis based on mask-conditional GANs for generating high-fidelity and diverse Cardiac Magnetic Resonance (CMR) images^9^. Although different GAN-based frameworks have been applied in the generation of medical images, they still need to improve the performance in the field of pathological images, and there are also few studies on the generation of glomerular pathological images to improve segmentation performance.

### Deep learning on glomerular identification and classification

In recent years, an increasing number of methods based on Deep Learning have been proposed to realize the identification and classification of glomeruli in digital pathological images. Each of these approaches has its own advantages and drawbacks. Jon N. Marsh et al. used a fully convolutional neural network based on the VGG16 architecture for glomerular segmentation and achieved 0.784 Aggregate Dice coefficients for nonglobally sclerosed glomeruli and 0.600 for globally sclerosed glomeruli^10^. Jaime Gallego et al. trained the Unet model on PAS-stained WSIs and H&E-stained WSIs. On the PAS-stained WSIs, normal and sclerosed glomeruli were classified with F1-scores of 97.5% and 68.8%, respectively. On H&E-stained WSIs, F1-scores of 90.8% and 78.1% were achieved^11^. Gloria Bueno proposed the sequential CNN segmentation-classification strategy(SegNet-AlexNet) and this two-step framework achieved 98.16% accuracy in classifying normal and sclerosed glomeruli when trained on 47 PAS-stained WSIs^12^. Lei Jiang et al. trained cascade mask region-based CNN architecture to detect, classify, and segment glomeruli into three categories: (i) GN, structural normal; (ii) global sclerosis; and iii) glomerular with other lesions. They achieved F1 scores of 0.839, 0.806, and 0.753, respectively, in the whole-slide image group^13^. Tianyuan Yao et al*.* developed and released a holistic Glo-In-One open-source toolkit to provide holistic glomerular detection, segmentation, and lesion characterization^14^. Kawazoe et al. developed an automated computational pipeline for detecting glomeruli on PAS-stained WSIs, followed by segmenting Bowman’s space, the glomerular tuft, the crescentic, and the sclerotic region inside of the glomeruli^15^. Silva et al. proposed the end-to-end network, named DS-FNet, combining the strengths of semantic segmentation and semantic boundary detection networks via an attention-aware mechanism, and it showed consistently high performance in a one-to-many-stain glomerulus segmentation^16^.

Most of the existing studies have not focused on the performance improvement of sclerosed glomerular segmentation. However, in fields such as kidney transplantation, the evaluation of sclerosed glomeruli is necessary and meaningful. Therefore, this paper focuses on improving the identification and segmentation performance of sclerosed glomeruli while solving the problem of automatic identification and segmentation of glomeruli.

## Materials

### Data source

In our study, we collected 51 WSIs from open sources, and they are introduced as follows. Thirty-one WSIs generated by the European project AIDPATH (http://aidpath.eu) were chosen. The tissue samples were stained using periodic acid–Schiff (PAS) and were scanned at 20× with a Leica Aperio ScanScope CS scanner^17^. The remaining 20 WSIs representing various human kidney pathologies came from four sources: three independent medical centres and TCGA. The data from three independent medical centres were collected by^10^, including 4 H&E-stained slides from the Military Institute of Medicine in Warsaw in Poland, six PAS-stained slides from Hospital Universitario Valld’Hebron, Barcelona in Spain and five H&E-stained slides from Cedars-Sinai Medical Center in Los Angeles in the USA. Five H&E-stained slides from the publicly available TCGA repository^18^. All slides were prepared from formalin-fixed paraffin-embedded (FFPE) sections with a thickness of 4 μm. The literature^11^ specifically describes the method followed for making the slides.

The 31 WSIs from^17^ contain two folders. DATASET_A: Raw data with 31 whole slide images (WSIs) in SVS format. We converted these to PNG format. DATASET_B: 2340 images with a single glomerulus, 1170 normal glomeruli and 1170 sclerosed glomeruli. All of them are in PNG format and are detected from DATASET_A. As the repository only provided normal glomerular patches and sclerosed glomerular patches, with the help of professional pathologists, we should find the specific locations of these glomeruli in WSIs, and use QuPath software to label categories and draw their outlines. For 20 WSIs from three independent medical centres and TCGA, the pathologists in^11^ first identified 78 ROIs and then delineated 1,184 glomeruli within the ROIs. ROIs were extracted for × 10, which corresponded to a pixel size of ~ 10 μm. In the availability of materials and data section of this article, we provide the URL where the data can be obtained.

### Data processing

**Figure 1:**
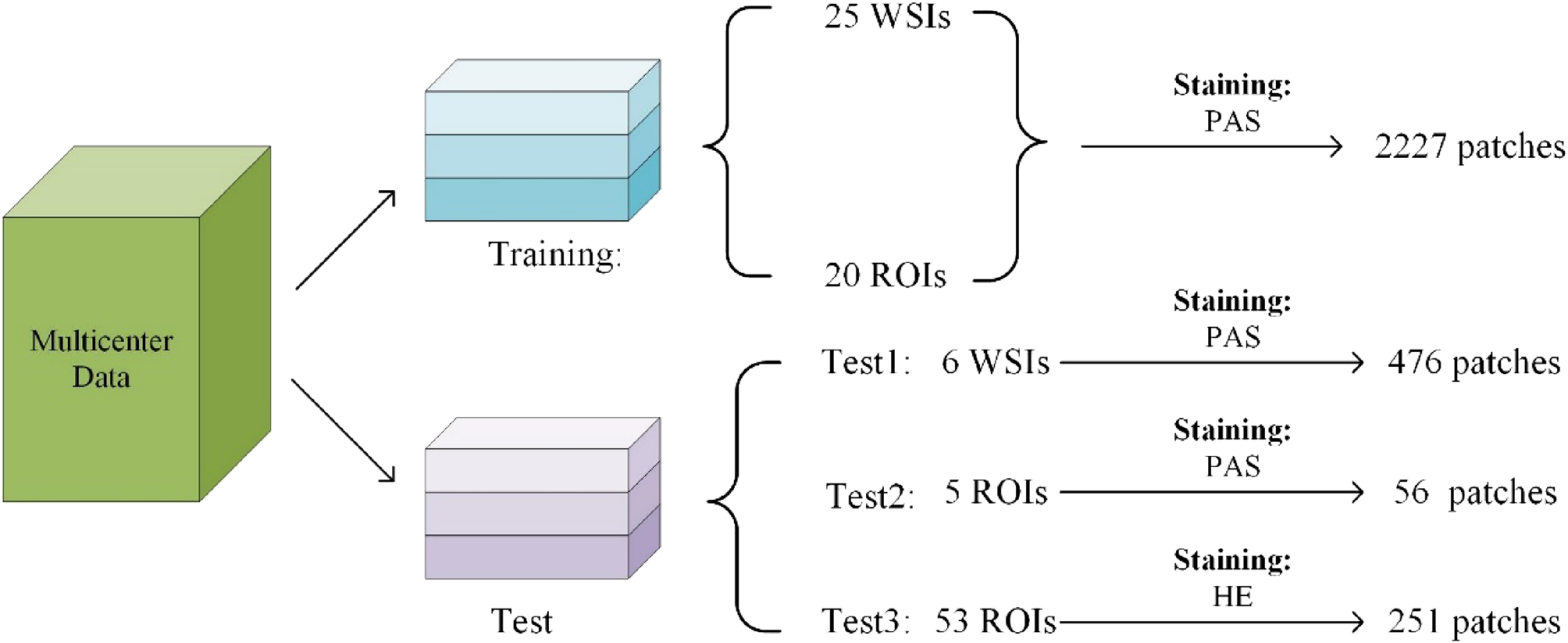
Data processing and settings.

**Figure 2:**
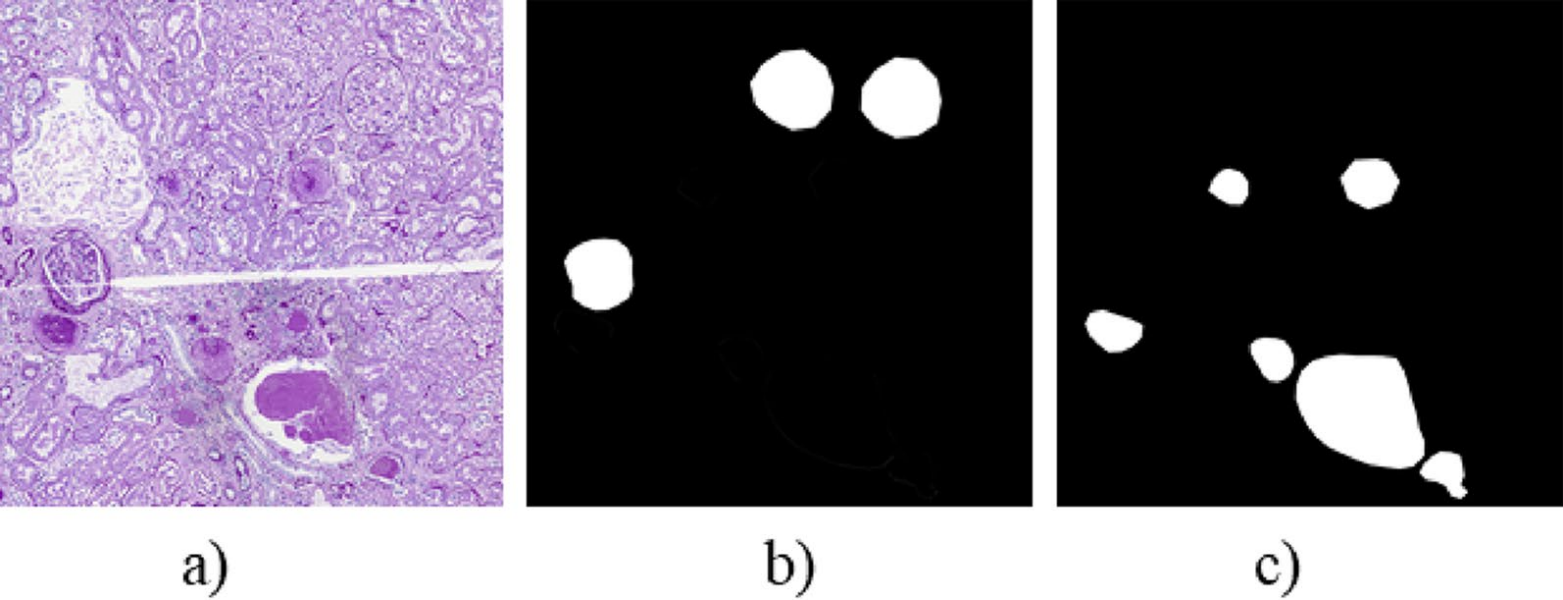
Label making. (**a**) Original patch, (**b**) normal glomerular mask, (**c**) sclerosed glomerular mask.

We used 25 PAS-stained WSIs from AIDPATH and 20 PAS stained ROIs from three independent medical centres and TCGA as training data for glomerular identification and classification, and the remaining six WSIs from AIDPATH and 58 ROIs were used as different test sets to verify the effectiveness of the glomerular identification algorithm. As shown in Fig. 1, we called the six PAS-stained WSIs from AIDAPTH Test1, the five PAS-stained ROIs from Zenodo and the 53 H&E-stained ROIs from Zenodo Test2 and Test3. Because the training data did not include H&E-stained slides, we could also test algorithm migration performances on H&E- stained WSIs. Since the resolution of a single WSI or ROI was very high, it was not easy to train. We performed overlapping cropping on WSIs or ROIs. The size of the cropping was 1024 × 1024, and the step length was 512. For the test sets, we also adopted the same strategy as the processing method of the training sets. The number of patches obtained from different datasets is shown in Fig. 1. To reduce the training and testing time, we downsampled all slides two times to reduce the size of the picture before cropping. We converted all glomerular contour labelling into pixelwise mask. Specifically, each WSI corresponded to two masks, with black representing the background and white representing all the normal glomeruli and sclerosed glomeruli, respectively. Figure 2 shows the masks of normal and sclerosed glomeruli on a patch.

## Methods

### Framework of sclerosed glomerular generation

#### Cropped sclerosed glomerular masks

**Figure 3:**
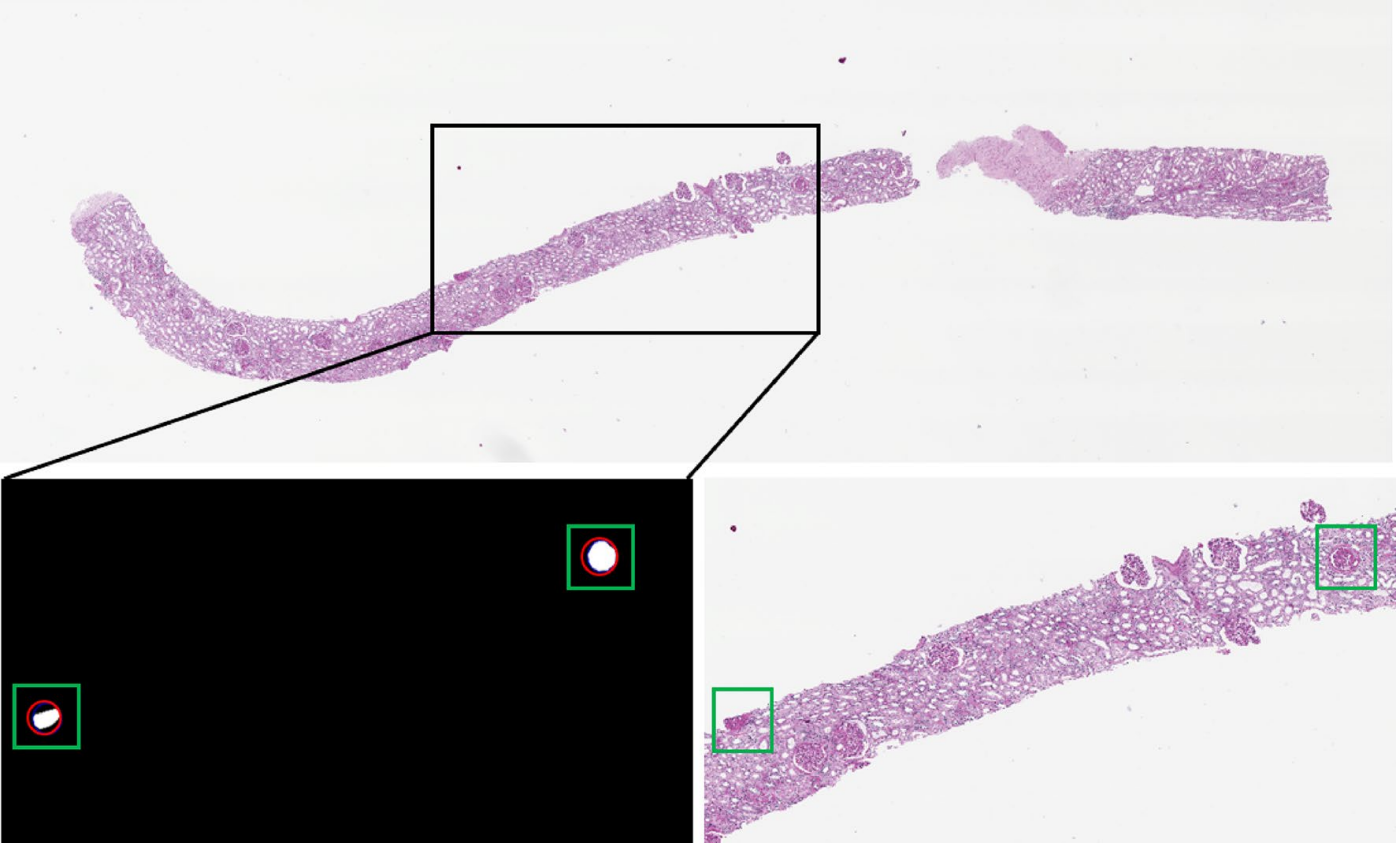
Explanation of the sclerosed glomerular cutting method.

To realize the generation of sclerosed glomeruli considering shapes and contextual information, we must create sclerosed glomerular datasets and corresponding masks to train the image inpainting network. Inspired by^19,20^, we synthesized our datasets using existing data. Specifically, the datasets were created as shown in Fig. 3. It is worth noting that our cropping method is designed to place the sclerosed glomeruli in the centre of the cropped image as much as possible, thus potentially assisting in the subsequent training of the generative network.

**Figure 4:**
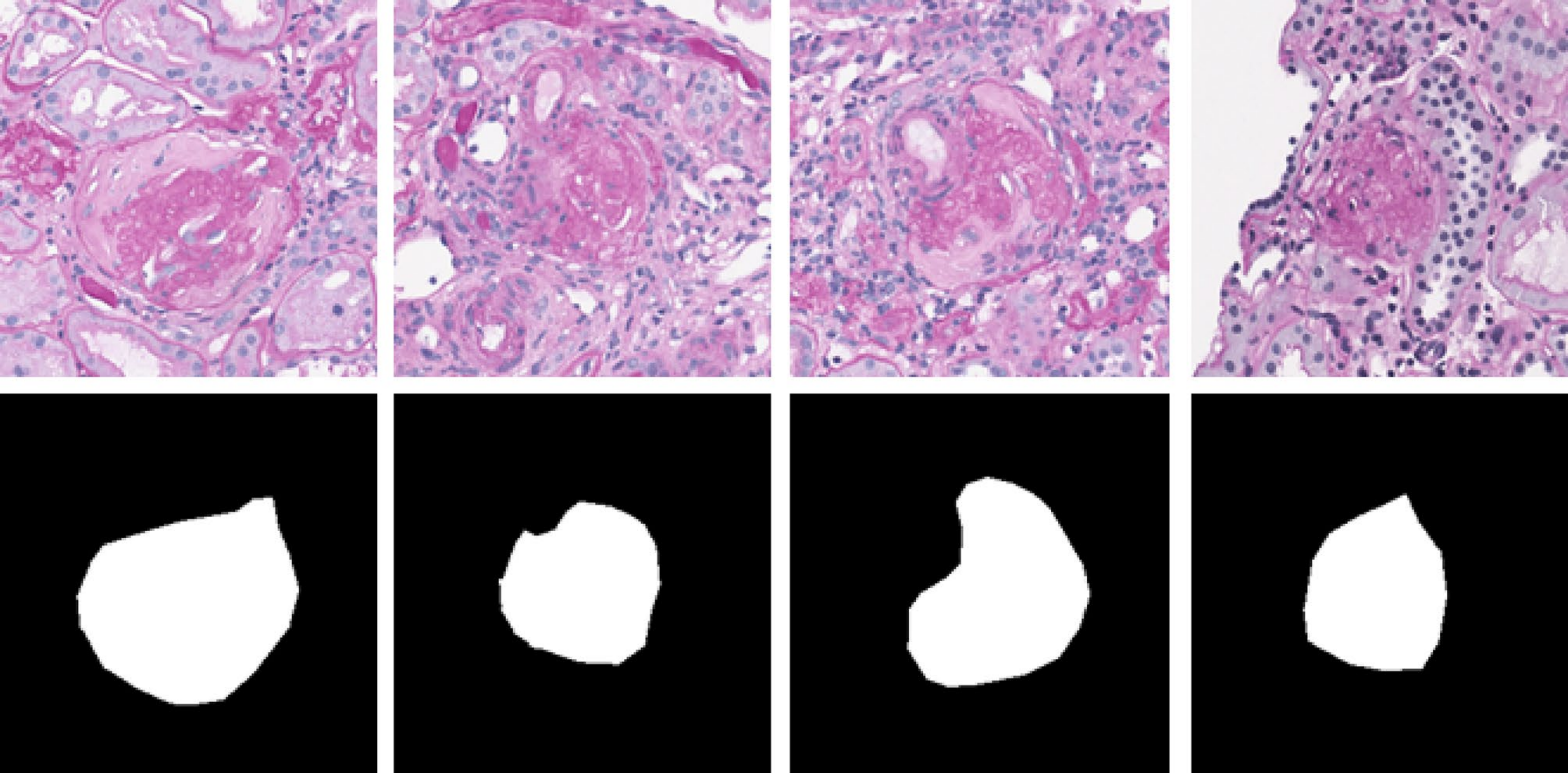
Illustration of cropped sclerosed glomerular images (1st row) and their corresponding images with sclerosed glomerular masks (2nd row).

First, we need to obtain all sclerosed glomerular masks from the segmentation training set. In Fig. 3, the bottom left portion shows the mask of a certain part of sclerosed glomeruli that is shown in the full section image (Fig. 3 top half). Based on the sclerosed glomerular labels provided by the masks of the open data source, the minimum peripheral circle was made for each sclerosed glomerulus, as shown in the red circles in the bottom left of Fig. 3. The centre of the outer circle was taken as the centre of the cropped rectangle picture, and the size is $256\times 256$, as shown in the green rectangle box in the bottom left section. The position of the rectangular box is mapped to the position of the original slice, as shown in the bottom right of Fig. 3. The rectangular masks and the corresponding pictures were cropped. The final training data are shown in the Fig. 4.

The ROIs are usually synthesized in both the foreground and the background to be fair and unbiased. For example, in^21,22^, the authors adopted this idea for synthesis. However, when considering the synthesis of ROIs in this article, we did not consider the generation both the glomeruli and the adjacent tissues (background). The reasons are as follows. In the segmentation task, the area of the sclerosed glomerular regions is relatively small compared to the area of its background. Thus, when the deep learning network segments and classifies the glomeruli in the image, the fraction of other parts is much higher than the fraction of glomerular regions. So, the diversity of other tissues can be guaranteed. Based on this, we adopted a generation way like image inpainting to make the generated glomeruli have a good fusion with the existing adjacent tissue and reduce the number of training parameters. Subsequently we can also generate the adjacent tissue and combine it with the generated glomeruli, which may make our model more robust.

#### Architecture of sclerosed glomerular inpainting network

**Figure 5:**
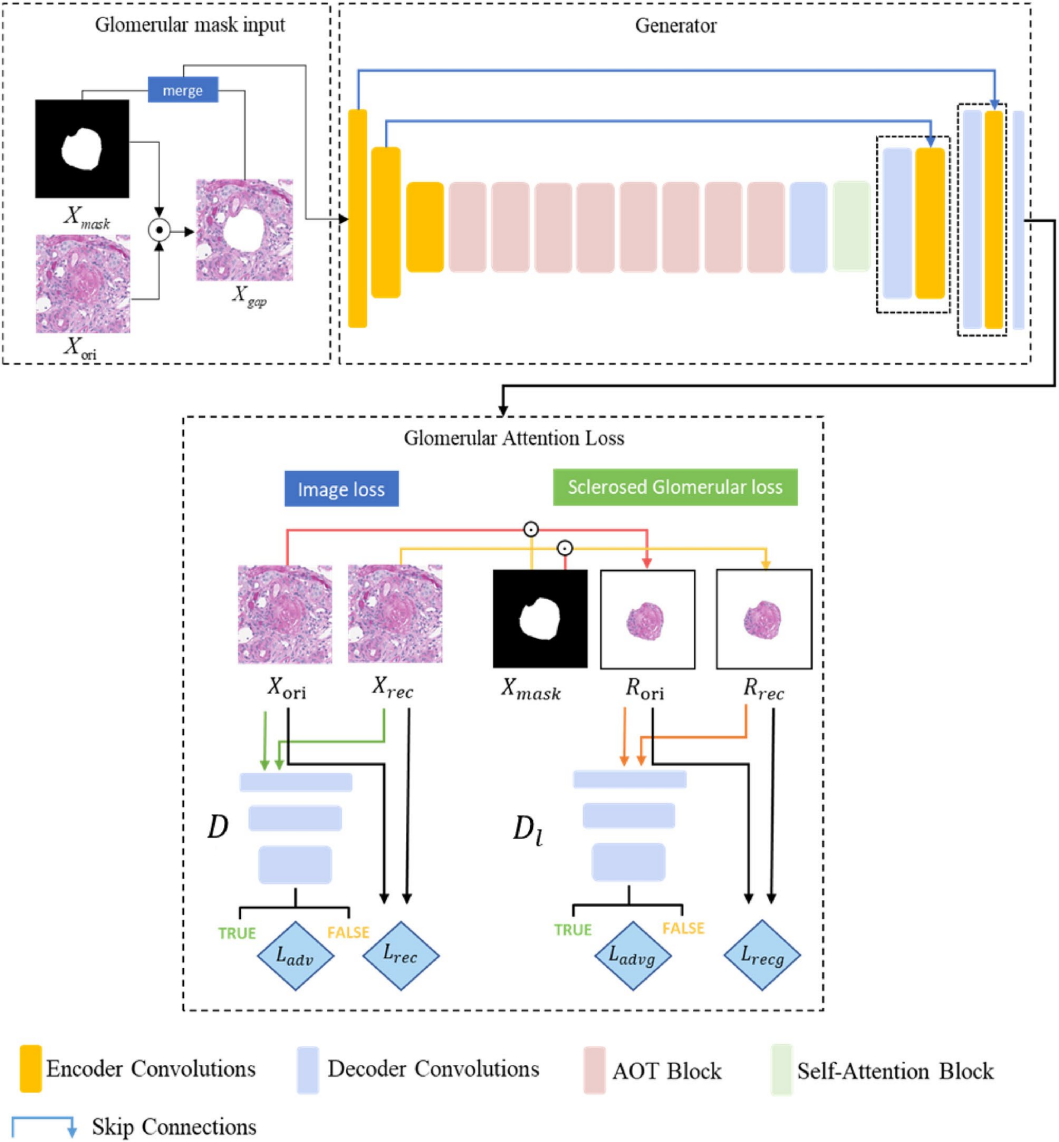
Architecture of sclerosed glomerular inpainting network.

The training phases of the sclerosed glomerular inpainting network are shown in Fig. 5. It is divided into four modules as follows. (1) The sclerosed glomerular mask input module controls the area of sclerosed glomerular generation. (2) The Generator module is mainly based on AutoEncoder, which consists of an encoder and a decoder. (3) The discriminator module mainly determines whether the input picture is a real picture or a generated picture and, in turn, promotes the training of the generator. (4) The sclerosed glomerular attention loss module includes the global image loss and the loss of the sclerosed glomerular foreground itself.

### A. Sclerosed glomerular mask input

A picture with only background ${X}_{{\text{gap}}}$ is obtained by Eq. (1), where ${X}_{{\text{ori}}}$ represents a cropped picture of the sclerosed glomerulus with background,${X}_{{\text{mask}}}$ represents the corresponding masks, and ⊙represents pixelwise multiplication. According to Eq. (2), we can obtain the network input ${X}_{{\text{input}}}$ by merging the images ${X}_{{\text{gap}}}$ and the masks ${X}_{{\text{mask}}}$ in the channel dimension, where $merge(\bullet )$ is the function realizing dimension concatenation. Through passing ${X}_{{\text{input}}}$ into the generator, we can realize sclerosed glomerular generation at the vacancy.

1 $$\begin{array}{c}{{X}_{{\text{gap}}}=X}_{{\text{ori}}}\odot \left(1-{X}_{{\text{mask}}}\right)\end{array}$$

2 $${{X}_{{\text{input}}}=merge(X}_{{\text{gap}}}{,X}_{{\text{mask}}})$$

### B. Generator

**Figure 6:**
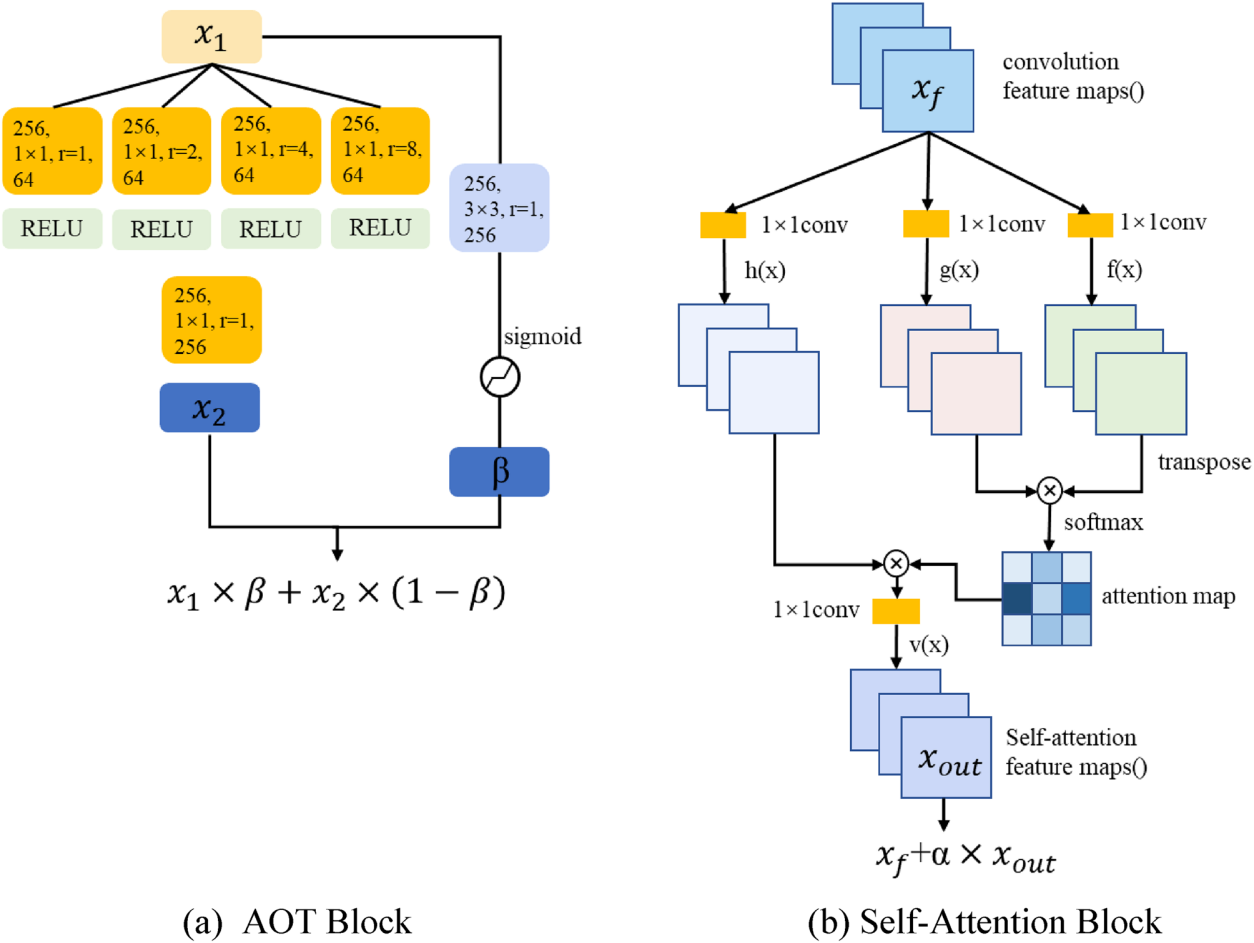
Key modules in the inpainting network. (**a**) Architecture of the AOT block. (**b**) Architecture of the self-attention block.

The generator consists of an encoder, a stack of building blocks, a self-attention block and a decoder. In addition, we use skip connections between the encoder and the decoder. The generator takes the 256 × 256 ${X}_{{\text{input}}}$ as the input. In the encoder section, the input first passes through a convolutional network of 7 × 7 convolution kernel size, with batch normalization and a LeakyReLU activation function, followed by two 4 × 4 convolutional layers with a stride of 2 to downsample the image. This is followed by eight AOT blocks, all with the same parameter settings to reduce the amount of computation required. The AOT block was proposed in^23^, and the architecture is shown in Fig. 6a. AOT blocks adopt the split-transformation-merge strategy in three steps^24^. During the transformation, each subkernel performs a different transformation of the input feature ${x}_{1}$ by using a different dilation rate. Inspired by ResNet, a gated residual connection first calculates the spatially-variant gate value $\upbeta$ from × 1 by a standard convolution and a sigmoid operation, and then the AOT block aggregates the input feature × 1 and the learned residual feature × 2 by a weighted sum with $\upbeta$.The network structure of the decoded part and the encoding part are consistent, and two deconvolution layers are used to make the size of the masked picture the same as the size of the input image. Before the first layer of the upsampling network, there is a self-attention block whose input size is $64\times 64$. It is proposed in^25^. As shown in Fig. 6b, by obtaining the self-attention feature maps, we can explore the relationship between the locality of the picture and the whole to solve the problem of long-distance dependence. Finally, the tanh function is applied in the output layer.

### C. Discriminator

The discriminator in this task was set to two, namely, the local discriminator and the global discriminator. The local discriminator only discriminated the generated sclerosed glomeruli, and the global discriminator discriminated the complete generated images, including the sclerosed glomeruli and the background. When we used the local discriminator, the region of the nonglomerulus can be filled in white so that the size of the local glomeruli image is consistent with the input whole image. In this way, the local discriminator has the same network structure as the global discriminator to reduce the amount of calculation. The input size of each discriminator is $256\times 256\mathrm{ pixels}$. There are a total of six convolutional layers, and each convolutional layer uses a 4 × 4 kernel with a stride of 2 (Convolution + LeakyReLU + Batch normalization) to decrease the size of the feature representations. The number of channels in the discriminator is set to 64, 128, 256, 512 and 1. The last layer of both discriminators produces N × N output patches representing classification scores (‘real’ or ‘fake’).

### D. Sclerosed glomerular attention loss module

As shown in Fig. 5, the image loss and sclerosed glomerular loss are set up to ensure that the whole pictures remain consistent and that the sclerosed glomeruli show a sense of clear texture and staining. Based on the designed loss module with the nature of attention, the generated network achieved a balance between the generation of glomeruli itself and the inpainting of the complete image.

The adversarial loss of the global image and local sclerosed glomeruli are shown in Eqs. (3) and (4), respectively, where D is the global discriminator and ${D}_{l}$ is the local discriminator. To reduce the amount of computation required, we set the network parameters of D and ${D}_{l}$ to be the same. ${X}_{{\text{rec}}}$ is the generated global image, which is obtained by the generator as shown in Eq. (5), where G represents the generator. ${R}_{ori}$ and ${R}_{rec}$ are the original local sclerosed glomeruli and the generated local sclerosed glomeruli, respectively. They are obtained as shown in Eqs. (6) and (7).

3 $$\begin{array}{c}{L}_{{\text{advg}}}=E\left[D{\left({X}_{{\text{rec}}}\right)}^{2}\right]+E\left[{\left(1-D\left({X}_{{\text{ori}}}\right)\right)}^{2}\right]\end{array}$$

4 $$\begin{array}{c}{L}_{{\text{advl}}}=E\left[{D}_{l}{\left({R}_{{\text{rec}}}\right)}^{2}\right]+E\left[{\left(1-{D}_{l}\left({R}_{{\text{ori}}}\right)\right)}^{2}\right]\end{array}$$

5 $$\begin{array}{c}{X}_{{\text{rec}}}=G\left({X}_{{\text{input}}}\right)\end{array}$$

6 $$\begin{array}{c}{R}_{ori}= {X}_{{\text{ori}}}\odot {X}_{{\text{mask}}}+\left(1- {X}_{{\text{mask}}}\right)\end{array}$$

7 $$\begin{array}{c}{R}_{rec}= {X}_{{\text{rec}}}\odot {X}_{{\text{mask}}}+\left(1- {X}_{{\text{mask}}}\right)\end{array}$$

Usually, in the field of image generation, we use pixel reconstruction loss (${L}_{1}$) to describe the pixel difference between images. As shown in Eqs. (8) and (9), ${L}_{1g}$ and ${L}_{1l}$ represent global ${L}_{1}$ and local ${L}_{1}$.

8 $$\begin{array}{c}{L}_{1g}=\parallel {X}_{{\text{ori}}}-{{X}_{{\text{rec}}}\parallel }_{1}\end{array}$$

9 $$\begin{array}{c}{L}_{1l}=\parallel {R}_{{\text{ori}}}-{{R}_{{\text{rec}}}\parallel }_{1}\end{array}$$

With the good effect of the generative algorithm in the field of image style transformation, the image features extracted by the convolutional network have been widely used as part of the objective function. We use the perceptual loss and style loss of the global image, which are shown in Eqs. (10) and (11), respectively.

10 $$\begin{array}{c}{L}_{per}=\sum_{i=1}^{{N}_{i}}\frac{{\parallel \left.{\phi }_{i}({X}_{{\text{ori}}})-{\phi }_{i}({X}_{{\text{rec}}}\right)\parallel }_{1}}{{N}_{i}}\end{array}$$

where ${\phi }_{i}$ is the activation map from the *i*-th layers of a pretrained network (e.g., VGG19^26^)$\mathrm{and } {N}_{i}$ is the number of elements in ${\phi }_{i}$. Similarly, the style loss is defined as the *L*1 distance between the Gram matrices of deep features of inpainting and real images.

11 $$\begin{array}{c}{L}_{sty}={\mathbb{E}}_{i}\left[{\parallel \left.{\phi }_{i}{\left({X}_{{\text{ori}}}\right)}^{T}{\phi }_{i}({X}_{{\text{ori}}})-{\phi }_{i}{\left({X}_{{\text{rec}}}\right)}^{T}{\phi }_{i}({X}_{{\text{rec}}}\right)\parallel }_{1}\right]\end{array}$$

The loss values of each part are added by a certain weight to obtain the final loss function, as shown in Eq. (12).

12 $$\begin{array}{c}{L}_{total}={\lambda }_{adv}{(L}_{{\text{advg}}}+{L}_{{\text{advl}}})+{\lambda }_{1g}{L}_{1g}+{\lambda }_{1l}{L}_{1l}+{\lambda }_{per}{L}_{per}+{\lambda }_{sty}{L}_{sty}\end{array}$$

where ${L}_{total}$ is the total loss and ${\lambda }_{adv}$=0.02, ${\lambda }_{1}$=1, ${\lambda }_{per}$=0.1, and ${\lambda }_{sty}$=150.

#### Process of synthesizing datasets

**Figure 7:**
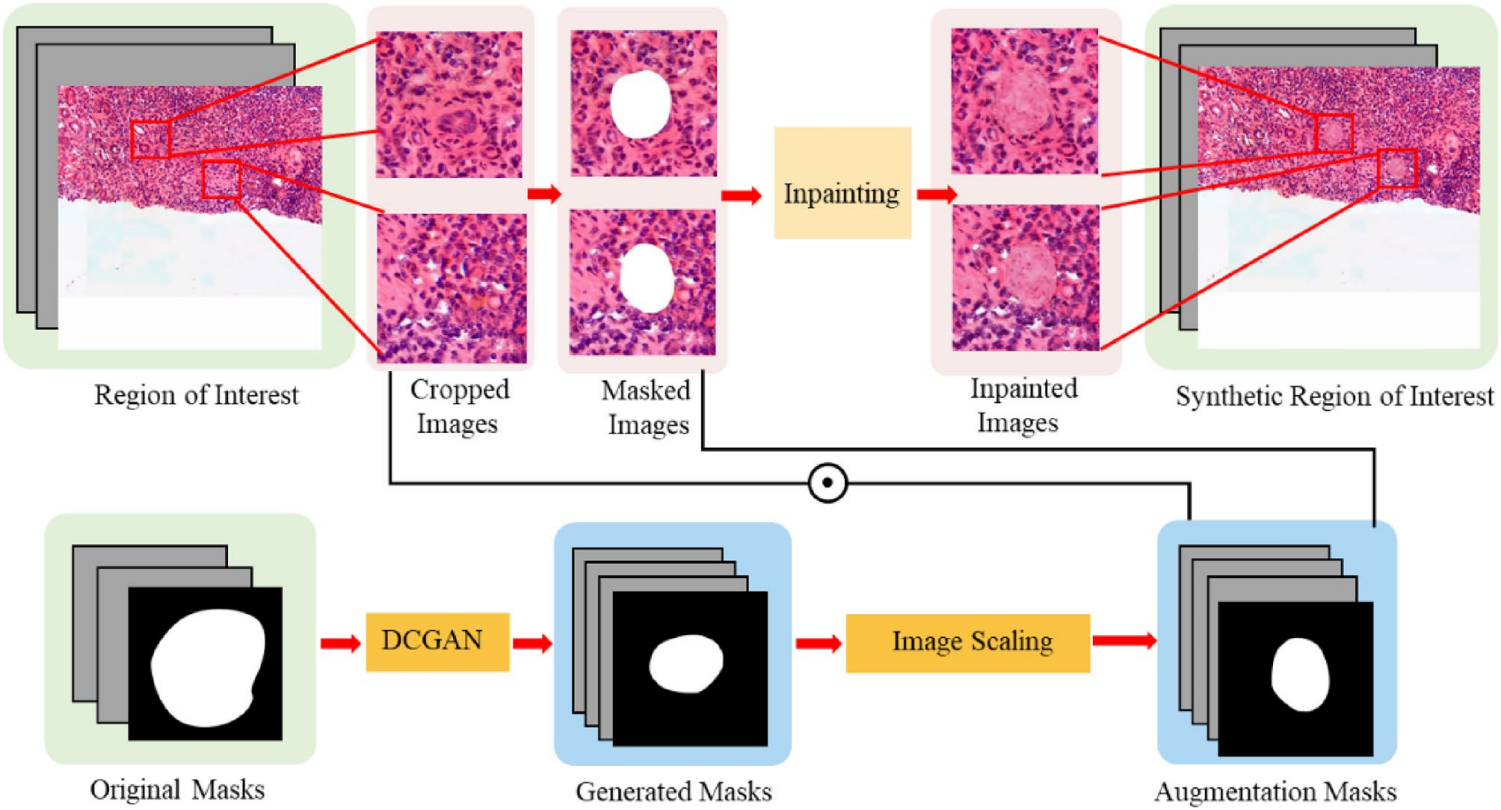
Testing phases of glomerular synthesis.

As shown in Fig. 7, in the stage of sclerosed glomerular synthesis, we used Deep Convolutional Generative Adversarial Network (DCGAN)^27^ to generate masks of different shapes and sizes based on the existing masks. Since colourful pixel values are likely to appear during mask generation, it is necessary to grayscale the generated masks and set a threshold at the same time to eliminate isolated regions in the masks whose area was smaller than the threshold. The glomerular contours in the masks are scaled so that the number of contours of different sizes are evenly distributed. Based on the pathologist's recommendation, we locate the potential positions for sclerosed glomeruli and cropped out squares of $256\times 256$ in these positions. Similar to the operation during training, masked images (${X}_{{\text{gap}}\_{\text{t}}}$) are obtained by pixelwise multiplication, as shown in Eq. (13) based on randomly selected generated masks (${X}_{{\text{gmask}}}$) and cropped images (${X}_{{\text{ori}}\_{\text{t}}}$).${X}_{{\text{gmask}}}$ and ${X}_{{\text{gap}}\_{\text{t}}}$ are concatenated as the input of the inpainting model. Finally, the generated images were merged into the original cropped area, and a new ROI with several sclerosed glomeruli in different positions was obtained.

13 $$\begin{array}{c}{{X}_{gap\_t}=X}_{{\text{ori}}\_{\text{t}}}\odot \left(1-{X}_{{\text{gmask}}}\right)\end{array}$$

### Glomerular segmentation network

**Figure 8:**
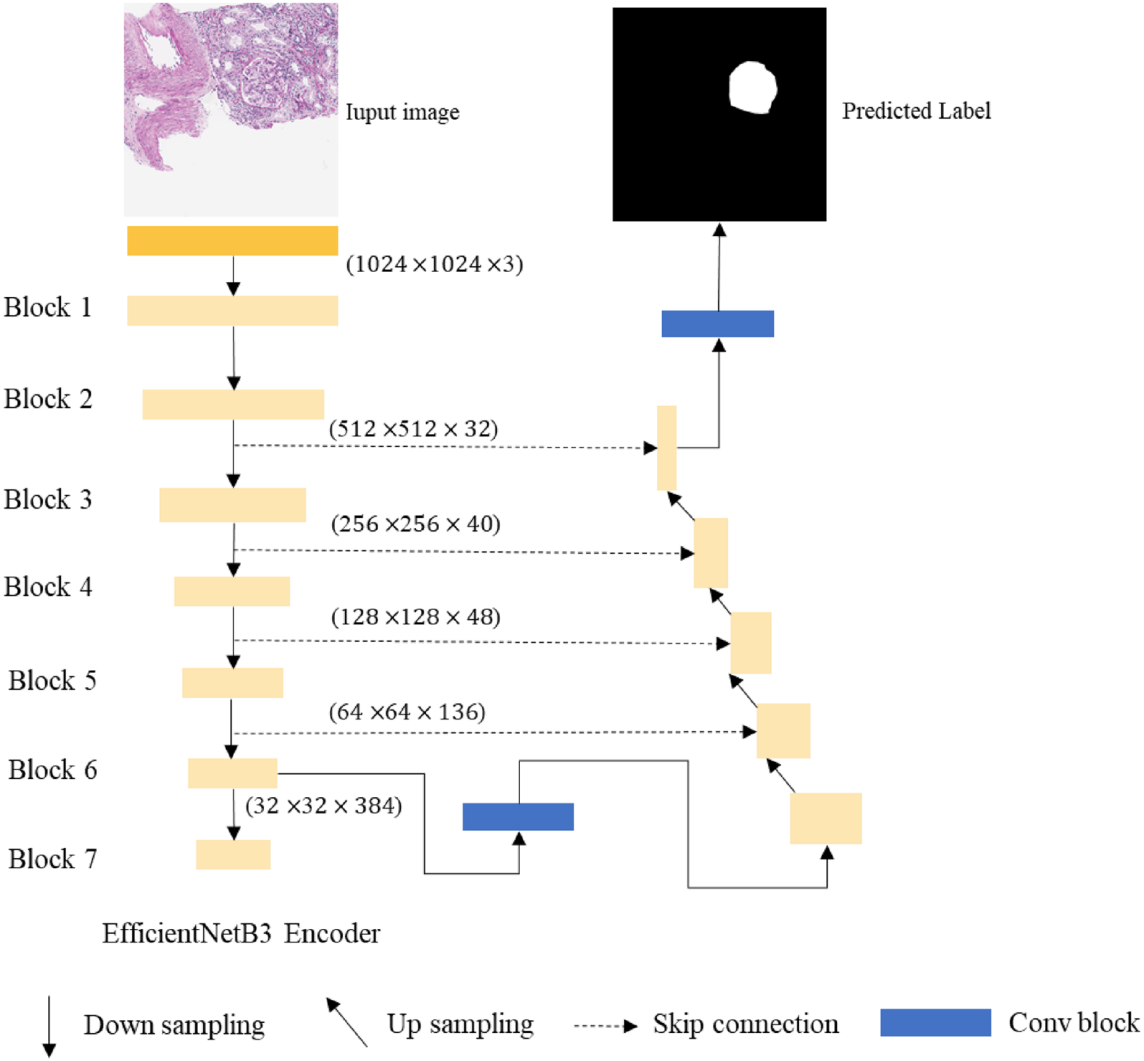
Architecture of the glomerular segmentation network.

For the design of the glomerular image segmentation network, we generally adopt an encoder-decoder architecture, within which the decoder structure is Unet. The skip connection in Unet is used to fuse multiscale features from the encoder with upsampled features. Here, shallow features and deep features are connected together to reduce the spatial information loss caused by downsampling. In the encoder part, we select EfficientNet as our encoder backbone. The reason why the more advanced transformer structure is not adopted here is that its performance heavily relies on pretraining and requires a large amount of computation. Thus, its training time and computation time will be higher than those of the CNN model under the same parameters. Meanwhile, its prediction time will be longer. We hope that EfficientNet can obtain the result faster while ensuring the effect, which is very important for slice evaluation. EfficientNet was proposed in^28^ and takes into account both the depth and width of the network. There are currently several versions of EfficientNet including B0-B7. To meet the speed and accuracy requirements of network training, we use EfficientNetB3 as the backbone of our encoder. The input size of the network is 1024 × 1024. Before training the model, the inputs are normalized. The network architecture is shown in Fig. 8.

When training the segmentation model, the batch size was 8, and the optimizer was Adam. We adopted CosineAnnealingLR in the Pytorch framework for the learning strategy and the minimum learning rate was 0.00001. We used the weighted sum of Binary CrossEntropy Loss (${L}_{BCE}$)and Dice Loss (${L}_{Dice}$)^29^ as the total loss (${L}_{seg}$). The specific formula is shown in Eq. (14), where $\lambda$ = 0.5. We trained our segmentation models on a single Tesla P100 (16 GB). A total of 200 epochs were trained, and the ratio of training sets and validation sets was 8:2. When evaluating the test set, the model with the lowest Dice coefficient of the validation sets was selected for evaluation.

14 $$\begin{array}{c}{L}_{seg=\lambda {L}_{BCE}+{L}_{Dice}}\end{array}$$

## Experiments and results

### Details and evaluation methods

Based on the images of sclerosed glomeruli and the corresponding masks obtained in the previous chapter, we trained and tested the inpainting network. The ratio of the training set to the test set was 8:2. We trained the models on a single Tesla P100 (16 GB). Here, we used ADAM as the optimizer with an initialized learning rate of 0.0001 and betas of {0.5; 0.999}. We trained our model for 100 epochs with a batch size of 16. The size of the network output was 256 × 256.

To characterize the model’s glomerular segmentation ability, especially the performance on sclerosed glomeruli, quantitative evaluation is needed. In the segmentation process, for each pixel in the image, there are two categories: positive and negative. If the prediction of positive or negative is correct, it is TP or TN. Conversely, it is FP or FN. Based on these four values, we can also obtain other commonly used metrics, as shown in Table 1.

**Table 1:** Equations of metrics performance.

Metric	Equation
Recall	$\frac{TP}{TP+FN}$
Precision	$\frac{TP}{TP+FP}$
F1-score	$2\times \frac{Precision\times Recall}{Precision+Recall}$
Dice	$\frac{2\times TP}{FP+2\times TP+FN}$

### Generation results and analysis

To evaluate the quality of generated sclerosed glomeruli quantitatively, we used the mean absolute error (MAE), peak signal-to-noise ratio (PSNR) and structural similarity index (SSIM) to compare the differences between generated glomeruli and the original. Table 2 shows the values of the three metrics.

**Table 2:** Values of inpainting performance.

Metric	Value
MAE	0.0152
PSNR	22.6943
SSIM	0.8086

**Figure 9:**
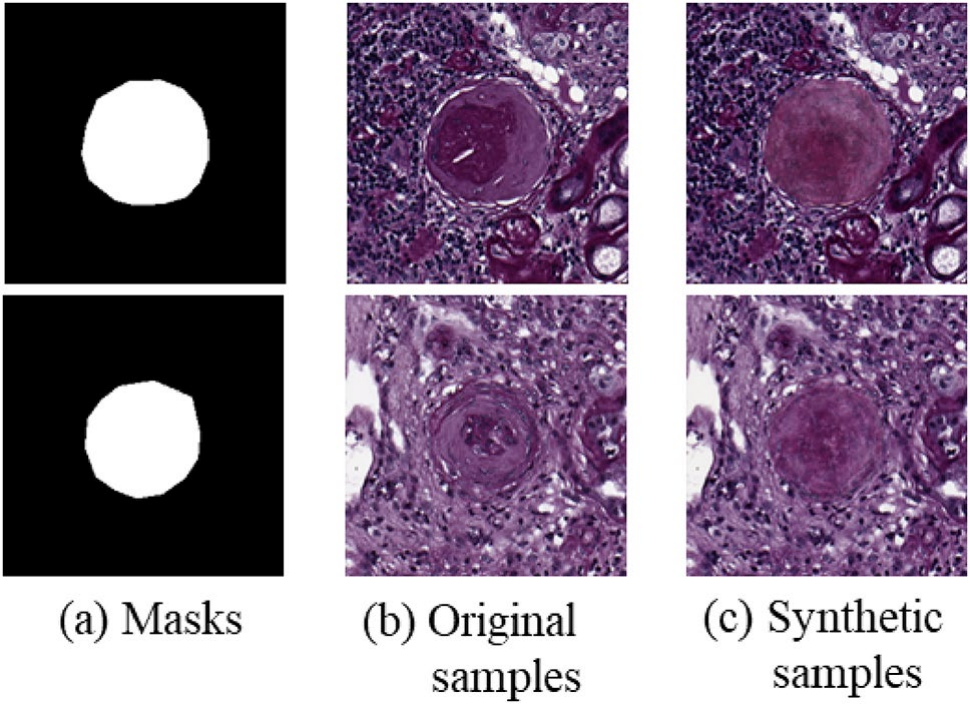
Examples of synthetic sclerosed glomeruli. (**a**) Sclerosed glomerular masks, (**b**) original samples, (**c**) synthetic samples.

As shown in Fig. 9, we obtained the synthetic images by combining the cropped glomeruli from the generated images with the background of the original images.

### Glomerular segmentation and analysis

Before adding generated sclerosed glomeruli to the training data, we verified the effect when using traditional data augmentation, including random flipping and rotating. We evaluated the performance of glomerular segmentation with traditional data augmentation and without traditional data augmentation for three test sets. The data augmentation strategy we adopted is an online method and each input training picture has a certain chance to be flipped or rotated. We performed ten experiments, with each experimental training set and validation set randomly divided. As shown in Table 3, we obtained the performance of glomerular segmentation based on our segmentation model under traditional data augmentation. “√” represents the use of traditional data augmentation.

**Table 3:** Performance of glomerular segmentation based on our segmentation model under traditional data augmentation or not, where NG-normal glomeruli and SG-sclerosed glomeruli.

	Trad aug	Class	Precision	Recall	F1	Dice
Test1 (PAS)	–	NG	0.8952 ± 0.0018	0.9169 ± 0.0031	0.9059 ± 0.0010	0.9036 ± 0.0005
		SG	0.8492 ± 0.0020	0.4089 ± 0.0025	0.5520 ± 0.0014	0.5145 ± 0.0006
	✔	NG	0.9028 ± 0013	0.9228 ± 0.0017	0.9127 ± 0.0006	0.9134 ± 0.0003
		SG	0.8451 ± 0021	0.5353 ± 0.0016	0.6534 ± 0.0009	0.6218 ± 0.0004
Test2 (PAS)	–	NG	0.9328 ± 0023	0.7727 ± 0.0020	0.8452 ± 0.0009	0.8445 ± 0.0004
		SG	0.7531 ± 0019	0.5577 ± 0.0028	0.6408 ± 0.0014	0.6355 ± 0.0007
	✔	NG	0.9202 ± 0.0030	0.7768 ± 0.0021	0.8424 ± 0.0011	0.8406 ± 0.0005
		SG	0.8409 ± 0.0032	0.5820 ± 0.0019	0.6879 ± 0.0012	0.6828 ± 0.0006
Test3 (HE)	–	NG	0.9725 ± 0.0028	0.6770 ± 0.0022	0.7983 ± 0.0011	0.7956 ± 0.0005
		SG	0.7496 ± 0.0029	0.2585 ± 0.0030	0.3844 ± 0.0023	0.3637 ± 0.0008
	✔	NG	0.9779 ± 0.0027	0.6871 ± 0.0021	0.8071 ± 0.0010	0.8052 ± 0.0005
		SG	0.7353 ± 0.0041	0.2334 ± 0.0032	0.3543 ± 0.0027	0.3439 ± 0.0010

The values represent the mean and standard deviation of the ten training times.

As seen from the Table 3, for different test sets, applying traditional data augmentation to training data can improve the overall effect of glomerular segmentation to a certain extent, but there may be a decline in precision. The experiments show that the segmentation performance of normal glomeruli is much better than that of sclerosed glomeruli, which is consistent with most of the current studies and validates the necessity of our data generation. At the same time, we see that the performance on test 3 is lower than that of test 1 and test 2, and we can conclude that the migration ability of the algorithm in recognition of renal pathological images with different staining needs to be improved because of the characteristics of different staining methods.

Therefore, in order to better evaluate the effect of the sclerosed glomerulus we generated on image segmentation, we analyse the influence of different amounts of synthetic data on the model identification ability for sclerosed glomeruli on the basis of traditional data augmentation, as shown in Table 4.

**Table 4:** Performance comparison of sclerosed glomerular segmentation based on our segmentation model when adding different amounts of synthetic data.

	Synthetic data	Precision	Recall	F1-score	Dice
Test1 (PAS)	+ 30%	0.8813 ± 0.0014	0.6025 ± 0.0038	0.7157 ± 0.0016	0.7010 ± 0.007
	+ 60%	0.8924 ± 0.0016	0.6426 ± 0.0032	0.7472 ± 0.0013	0.7328 ± 0.0006
	+ 100%	0.8947 ± 0.0021	0.6408 ± 0.0029	0.7468 ± 0.0012	0.7491 ± 0.0006
Test2 (PAS)	+ 30%	0.8825 ± 0.0023	0.6023 ± 0.0012	0.7160 ± 0.0008	0.7313 ± 0.0004
	+ 60%	0.9022 ± 0.0019	0.6219 ± 0.0027	0.7363 ± 0.0012	0.7641 ± 0.0006
	+ 100%	0.9068 ± 0.0025	0.6211 ± 0.0031	0.7372 ± 0.0014	0.7685 ± 0.0007
Test3 (HE)	+ 30%	0.7729 ± 0.0032	0.4017 ± 0.0042	0.5286 ± 0.0025	0.4278 ± 0.0009
	+ 60%	0.7761 ± 0.0029	0.4165 ± 0.0037	0.5421 ± 0.0022	0.4871 ± 0.0009
	+ 100%	0.7801 ± 0.0042	0.4303 ± 0.0025	0.5547 ± 0.0018	0.4916 ± 0.007

The values represent the mean and standard deviation of the ten training times.

Table 4 shows that by adding different amounts of synthetic data based on our algorithm, the segmentation performance of scleral glomerulus is greatly improved. This shows that the ability to recognize sclerosed glomeruli is improved, as our generated sclerosed glomeruli have different shapes and sizes distributed in different locations. However, the performance of adding more synthetic data is not always better than that of others. The reason for our analysis is that although the diversity of the generated data shapes is greatly improved, the mechanism features inside the sclerosed glomeruli are still generated based on the existing data, and the distribution of features is still consistent with the original ones.

Additionally, we compared our segmentation model with other classical models to verify the advantages of our method in the task of glomerular segmentation, as shown in Table 5.

**Table 5:** Performance comparison of glomerular segmentation based on three modes, including our proposed method.

	Model	Mean Precision	Mean Recall	Mean F1-score	Mean Dice
Test1 (PAS)	Unet	0.7223 ± 0.0012	0.6630 ± 0.0010	0.6914 ± 0.0006	0.7690 ± 0.0003
	Unet++	0.7816 ± 0.0014	0.6719 ± 0.0011	0.7226 ± 0.0006	0.7828 ± 0.0003
	Swin-Unet	0.8545 ± 0.0010	0.7730 ± 0.0014	0.8117 ± 0.0005	0.8025 ± 0.0003
	EfficientNetB3-Unet	0.9002 ± 0.0012	0.7918 ± 0.0009	0.8425 ± 0.0004	0.8325 ± 0.0002
Test2 (PAS)	Unet	0.8628 ± 0.0027	0.6423 ± 0.0012	0.7364 ± 0.0009	0.7310 ± 0.0004
	Unet++	0.8901 ± 0.0022	0.6310 ± 0.0027	0.7385 ± 0.0012	0.7524 ± 0.0006
	Swin-Unet	0.8804 ± 0.0008	0.6621 ± 0.0015	0.7556 ± 0.0006	0.7730 ± 0.0003
	EfficientNetB3-Unet	0.9137 ± 0.0020	0.6713 ± 0.0024	0.7740 ± 0.0010	0.8045 ± 0.0005
Test3 (HE)	Unet	0.7909 ± 0.0011	0.4871 ± 0.0020	0.6029 ± 0.0010	0.5521 ± 0.0004
	Unet++	0.8054 ± 0.0020	0.4993 ± 0.0017	0.6164 ± 0.0010	0.5564 ± 0.0004
	Swin-Unet	0.8621 ± 0.0029	0.5335 ± 0.0009	0.6591 ± 0.0009	0.6102 ± 0.0004
	EfficientNetB3-Unet	0.8790 ± 0.0018	0.5626 ± 0.0012	0.6861 ± 0.0007	0.6882 ± 0.0003

The values represent the mean and standard deviation of the ten training times.

Compared with other medical semantic segmentation algorithms including Unet and Unet++ ^30^ which are all trained with 100% generated data, we calculated the mean value of each metric of the two classes on the different test sets. We see that our algorithm performs better than other algorithms on different test sets. In addition, on the test set stained by H&E, our algorithm has greater advantages than the others, such as better generalization and migration ability.

**Figure 10:**
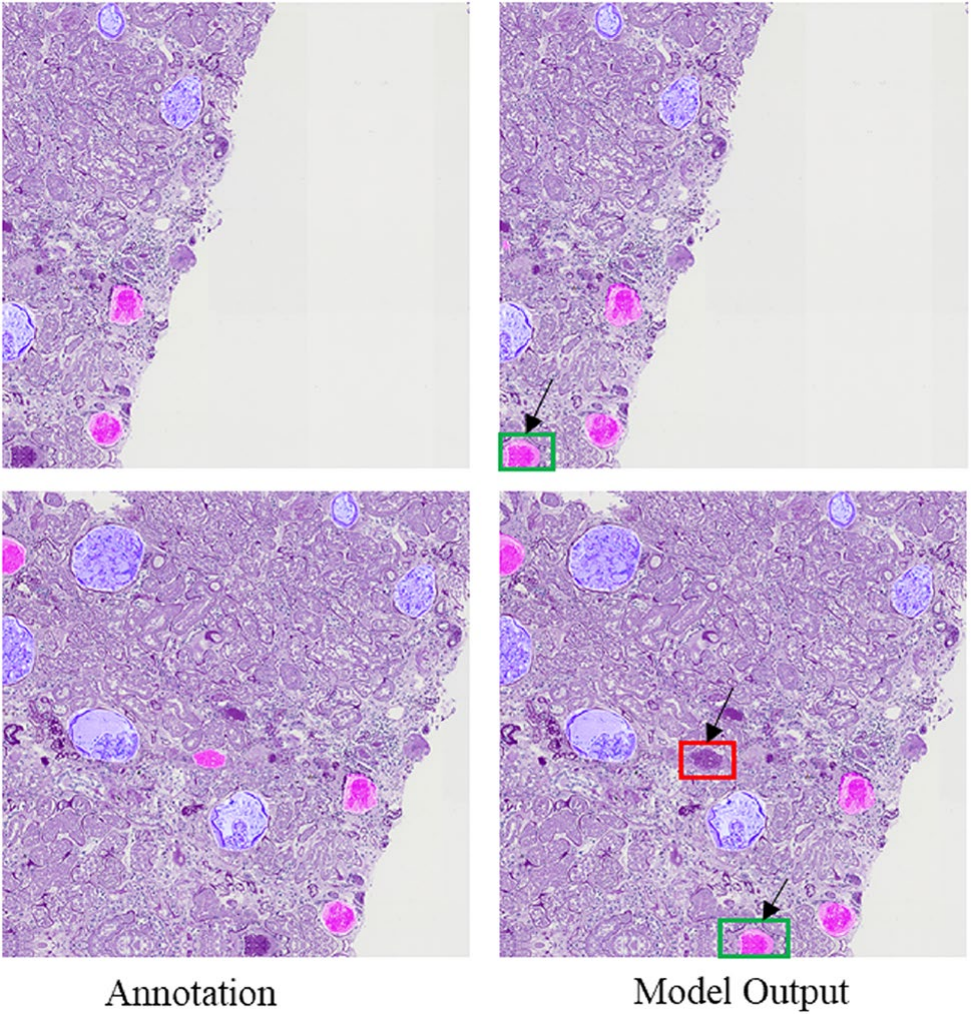
Segmentation results obtained by our model.

Figure 10 shows the visualization of our final model output, with the annotations of the data in the left column and the output of the model in the right column. Blue represents normal glomeruli, and red represents sclerosed glomeruli. We can see that our model can label sclerosed glomeruli that missed the mark in the original data label, especially in the marginal part, which shows the excellent identification ability of our model. However, at the same time, there are smaller sclerosed glomeruli that are missing and need to be improved.

## Conclusion

In the task of glomerular identification and classification, it is difficult and costly to obtain large amounts of data for training the model, and there is a problem of class imbalance because the number of sclerosed glomeruli is much larger than that of normal glomeruli in the available data. Therefore, we proposed a sclerosed glomerular generation method based on image inpainting. With the existing masks, we generated diverse masks and scaled them to obtain more small sclerosed glomeruli. By using the proposed image inpainting-based method and generated masks, we synthesized multiple images of sclerosed glomeruli with good fusion with the backgroundand ensured that the texture of sclerosed glomeruli was clear and true. We combined the synthesized data with the original data and passed them into the segmentation network. The glomerular segmentation network was based on Unet where we used EfficinetNetB3 as the backbone of the encoder. When we incorporated synthetic sclerosed glomeruli, we achieved better sclerosed glomerular identification under traditional data augmentation. Compared with other segmentation models, our model achieved the best mean F1 and Dice coefficients containing 2 classes by using EfficientNetB3-Unet.

Since our identification algorithm is mainly targeted at the training of globally sclerosed and normal glomeruli, the identification performance of other classes using our algorithm needs to be improved. Our generated glomeruli are also globally sclerosed, and we can further explore the controlled generation of different degrees of sclerosed glomeruli. In this way, we can reduce the number of missed glomerular tests.

## Data Availability

The 31 WSIs generated in the AIDPATH are hosted by Mendeley at: https://data.mendeley.com/datasets/k7nvtgn2x6/3 and 78 ROIs from 21WSIs are hosted by Mendeley at: https://zenodo.org/record/4299694.
